# Clinical Features of Children with COVID-19 in Initial Time of Pandemic

**DOI:** 10.14789/jmj.JMJ22-0005-OA

**Published:** 2022-12-14

**Authors:** TAKAHIKO HAYASHI, YAYOI MURANO, YAMATO MUTO, MICHIHIKO TAKASU, HIROYUKI SATO, HIROMI YAGISAWA, KARIN OSHIMA, NAO MIYAZAKI, TAKUYA ADACHI, KEN HISATA, TOMOYUKI NAKAZAWA, MAHBUBUR RAHMAN, STUART GILMOUR, TOSHIAKI SHIMIZU

**Affiliations:** 1 Division of Pediatrics, Toshima Hospital, Tokyo, Japan; 1 Division of Pediatrics, Toshima Hospital, Tokyo, Japan; 2 Department of Pediatrics, Juntendo University, Tokyo, Japan; 2 Department of Pediatrics, Juntendo University, Tokyo, Japan; 3 Graduate School of Public Health, St. Luke`s International University, Tokyo, Japan; 3 Graduate School of Public Health, St. Luke`s International University, Tokyo, Japan; 4 Division of Infectious Disease, Toshima Hospital, Tokyo, Japan; 4 Division of Infectious Disease, Toshima Hospital, Tokyo, Japan

**Keywords:** children, corona virus, COVID-19, SARS-CoV-2, pandemic

## Abstract

**Objectives:**

COVID-19 (Coronavirus Disease 2019) is now a global pandemic. Although children are said to have mild symptom, their clinical features are not known well. We conducted a retrospective study during initial term of pandemic to understand the difference of clinical features including clinical symptoms and patients’ characteristics of COVID-19 children and those without COVID-19.

**Materials:**

To compare clinical features between children with and without COVID-19, we collected data on children who received a COVID-19 test between March 25^th^ and October 31^st^, 2020. All data were collected from medical records.

**Methods:**

There were three groups of patients in the study sample; patients with COVID-19, patients with close COVID-19 contact and performed COVID-19 tests, and patients suspected COVID-19 but tested negative. We analyzed the clinical features of the groups.

**Results:**

A total of 108 patients were included in this study, of whom 30 were patients with COVID-19, 25 were patients with close COVID-19 contact, 51 were suspected COVID-19 but tested negative, and two were excluded because they were infants born from COVID-19 mothers. The statistical analysis showed that children with COVID-19 had contact with COVID-19 patients had fewer clinical symptoms including cough and fever compared to children with a negative test of COVID-19. Sensitivity analysis showed that fever, cough, fever and/or cough could not distinguish children with COVID-19 from those without COVID-19. As conclusion, children with COVID-19 have less symptoms as fever or cough and the clinical symptoms cannot distinguish them from children with other diseases.

## Introduction

COVID-19 (Coronavirus Disease 2019), an infection caused by SARS-CoV-2 (Severe acute respiratory syndrome coronavirus 2), has become a global pandemic. The pandemic has occurred in waves and gradually affecting younger age groups^1)^, whose symptoms are reported to be milder than in adults^2, 3)^, though the reasons remain unclear^4)^. Although their symptoms can be milder, children can be infected via family members and may also play an important role as transmission vectors^5, 6)^, so it is important to know the clinical features in child populations. Moreover, in clinical practice, it is important to distinguish children with potential COVID-19 from other diseases as an infection control strategy in outpatient clinics, where children without COVID-19 and their more vulnerable parents and elderly relatives may gather. However, there are few studies that compare child patients with and without COVID-19 for the purpose of developing infection control strategies.

We conducted a study to understand the clinical features of children with COVID-19 and compare them with outpatients without COVID-19. The study was conducted in Toshima hospital, which is the second largest hospital among 10 medical institutions for type 2 infectious disease with dedicated beds for infectious disease in Tokyo, and the largest hospital with pediatric COVID-19 patients.

## Material and Methods

We extracted data from medical records between March 25th 2020 and October 31st 2020 for all patients who attended the pediatric ward diagnosed with COVID-19 based on COVID-19 tests, children who had close contact with someone with COVID-19 who were recommended for COVID-19 testing by a public health center, and children who received a negative COVID-19 test from their attending pediatrician.

Data collected included date of arrival, age, sex, diagnosis, information about contact with COVID-19, and clinical symptoms including fever, cough, rhinorrhea, pneumonia and diarrhea. Among clinical symptoms, pneumonia was defined as an abnormal chest X ray finding or clinician diagnosis if no chest X ray was performed.

Children were divided into 3 groups: COVID-19 patients, close contacts who have COVID-19 patients in their family or close community such as school or nursery and were recommended for a COVID-19 test, and COVID-19-negative patients who had been suspected to have COVID-19. We used ANOVA to compare continuous variables (age) and chi-square tests to compare categorical variables (sex, contact with COVID-19 and clinical symptoms) between these groups. We calculated the sensitivity and specificity of symptoms including fever, cough, both fever and cough, and either fever or cough as a method for distinguishing the groups by symptoms.

Stata software version 15.1 was used.

The study was approved by the ethical committee of Toshima Hospital (No 2-28). Informed consent was difficult to obtain from the participants and the use of the data were informed to the guardians using documents with the opt-out approach.

## Results

A total of 108 patients were included in this study. There were 30 patients with a positive result for COVID-19. PCR (polymerase chain reaction) tests were conducted in 28 of these patients, a LAMP (Loop-mediated Isothermal Amplification) test in one, and an antigen test in one patient. There were 25 patients who had contact with COVID-19 and performed screening test as close contact patients but were negative on SARS-CoV-2 PCR, and 51 patients who were suspected to be COVID-19 positive by their attending pediatrician but confirmed negative on testing. We excluded 2 patients from this study because they were born from COVID-19 positive or suspected-positive mother.

Results of comparisons between three groups are shown in Table 1. There was no significant difference in age or sex between the groups. Compared to the children diagnosed with COVID- 19 or those who had contact with COVID-19, those who had negative SARS-CoV-2 results had significantly less contact with COVID-19 patients. Those with negative SARS-CoV-2 PCR results with COVID-19 had significantly more symptoms of cough and fever. Among children with COVID-19, there were 12 children without symptoms and among children with close contact, there were 13 children without symptom. There was no COVID- 19 child who met criteria of moderate or severe symptom.

**Table 1:** Results of comparisons between three groups

	COVID-19 (30)	Have contact but negative results (25)	COVID-19 was suspected but negative result (51)	p
Age (months)	80 ± 51.3	56.8 ± 51.0	63.9 ± 55.3	0.22
Sex				0.24
Boy (%)	15 (50.0)	10 (40.0)	24 (47.1)	
Girl (%)	15 (50.0)	15 (60.0)	27 (52.9)	
Contact with COVID-19				<0.05
Yes (%)	29 (96.7)	25 (100)	0 (0)	
No (%)	1 (3.33)	0 (0)	51 (100)	
Fever				<0.05
Yes (%)	14 (46.7)	6 (24.0)	44 (86.3)	
No (%)	16 (53.3)	19 (76.0)	7 (13.7)	
Cough				<0.05
	5 (16.7)	6 (24.0)	23 (45.1)	
	25 (83.3)	19 (76.0)	28 (54.9)	
Rhinorrhea				0.57
	9 (30.0)	6 (24.0)	10 (19.6)	
	21 (70.0)	19 (76.0)	41 (80.4)	
Diarrhea				0.92
	4 (13.3)	3 (12.0)	7 (13.7)	
	26 (86.7)	22 (88.0)	44 (86.3)	
Pneumonia				0.13
	1 (33.3)	0 (0)	6 (11.8)	
	29 (96.7)	25 (100)	45 (88.2)	
Any symptoms*				0.53
	18 (60.0)	12 (48.0)		
	12 (40.0)	13 (52.0)		

*: We excluded children who suspected COVID-19 but were not COVID-19 because they were suspected of COVID-19 because of fever.

Diagnoses for children with suspected COVID-19 but negative SARS-CoV-2 PCR results were upper respiratory tract infection (17), pneumonia (7), asthmatic bronchiolitis (5), febrile seizure (5), Kawasaki disease (3), lymphadenitis (3), gastroenteritis (2), urinary tract infection (2), appendicitis (1), subacute necrotizing lymphadenitis (2), fever of unknown origin (1), sun stroke (1), poor milk feeding (1), psychologic disorder who had prolonged fever for 1 weeks but finally diagnosed as psychologic fever (1).

Children with a COVID-19 diagnosis had contact with family (19), school (2), nursery (6), karate school (2), and unknown (1). Those who had contact with COVID-19 but a negative test result reported only contacts with family (23) and school (2).

Finally, we calculated the sensitivity and specificity of fever, cough, fever and cough, and fever or cough to distinguish COVID-19-positive and COVID-19-negative children. Sensitivity of fever, cough, fever and cough, and fever or cough are 0.45, 0.18, 0.09, and 0.54, respectively. Specificity of them are 0.15, 0.52, 0.63, and 0.03, respectively (Table 2).

**Table 2:** Sensitivity and specificity of symptoms

Symptoms	Sensitivity	Specificity
Fever	0.45	0.15
Cough	0.18	0.52
Fever and cough	0.09	0.63
Fever or cough	0.54	0.03

## Discussion

We conducted a study of COVID-19 in Japanese children at one of the largest admitting hospitals for COVID-19 patients in Tokyo. We could not find differences in symptoms between children with COVID-19 and children who had close contact but negative SARS-CoV-2 test results. On the other hand, children who were suspected to be COVID-19 patients but negative on SARS-CoV-2 PCR had more fever or cough symptoms compared to those with COVID-19. COVID-19 positive children had significantly more contact with COVID-19 patients whereas children with negative SARS-CoV-2 results had almost no contact with COVID-19 patients. Sensitivity and specificity of symptoms as fever and cough were low and are not useful to distinguish children with and without COVID-19.

It is well-known that children have lower susceptibility to SARS-CoV-2^3, 7)^, but most of the studies are comparisons between children and adults. However, in our daily pediatric practice, we need to know if the patient had COVID-19 or not in the child population, and Japanese Society for Infection Prevention and Control guidelines recommend to ask patients with COVID-19 symptoms to take distance from other patients when waiting at an outpatient clinic. However, in our study, we could not distinguish children with COVID-19 and without COVID-19 by clinical symptoms such as fever and cough which means that dividing patients in waiting rooms into COVID-19 suspected and non-suspected groups, clinical symptoms will not be effective, and separating children by presenting symptoms will result in mixing of both COVID-19 and non-COVID-19 patients in the same waiting areas. What we can do so far is to require personal protective methods in all children and medical staff even in general outpatient clinics throughout this pandemic.

The lack of symptoms in COVID-19 children in this study also raises significant concerns about the usefulness of passive or active case-finding to control the pandemic in children. While contact tracing is a known effective mechanism for controlling the pandemic and recommended by the WHO^8)^, in the absence of a mass testing program and with widespread community transmission, our study shows that COVID-19 children are unlikely to be identified based on symptoms alone, and will spread the disease widely until an adult is infected and presents with symptoms. While schools and nurseries remain open the pandemic cannot be controlled without mass testing, and the government needs to reconsider approaches to pandemic control based on symptomatic case finding alone. Years after the beginning of this pandemic the disease remains largely uncontrolled in Japan, and it is time to recognize that the Japanese government’s control efforts have been insufficient. A shift to widespread mass testing would overcome the challenges in identifying coronavirus in children, and enable the government to more rapidly control the disease, especially if it is to continue to keep schools open.

Our study suggests that among several contact pathways, contact with family members plays an important role in transmission, consistent with other studies^9)^. Among other contact situations, we found transmission in school, nursery and karate lessons. Although there were a small number of patients with these transmission routes, we must remain aware of them, as they are indicators of uncontrolled community spread. School children are educated to use masks, maintain social distance, and wash their hands but the effectiveness of these strategies outside the household has not been established^10)^, younger children are unable to wear masks^11)^ and activities that require physical contact like karate make these practices impossible to follow. Moreover, there are studies that report the presence of SARS-CoV-2 positive results in stool samples after recovery from COVID-19^12)^, so that such personal protective measures may be impossible in preschool children.

There are some limitations in our study. This study is the study done at the very first time of the pandemic and some results cannot be applied to the present situation. Our hospital only has a six- bed intensive care unit, and so severe cases are admitted to other hospitals. However, to the best of our knowledge, there was no severe pediatric case in Tokyo during the study period. Another limitation is that many positive cases are diagnosed at public health centers and children who were suspected to have COVID-19 and tested SARS- CoV-2 came to our hospital directly. Finally, we concluded that clinical symptoms do not distinguish children with and without COVID-19 based on sensitivity and specificity of clinical symptoms, however, we should consider that the prior priority differs between the groups. However, as we are currently in a pandemic situation, our study may be helpful for daily pediatric practice.

## Conclusion

We could not distinguish patients with COVID-19 and those without COVID-19 by symptoms in children with close contact. Among daily practice, fever and cough were less likely to be seen in children with COVID-19.

## Funding

none.

## Author contributions

Methodology was established by TH, MT, HS, and TA. TH, MT, and HS conceptualized the study. YM, YM, HY, KO, NM, MR, and SG performed the analysis. TH, YM, YM, HY, and KO wrote the original draft and NM, TA, KH, TN, MR, SG, and TS reviewed and edited it. TN, KH, MR, SG, and TS supervised this study. All authors read and approved the final manuscript.

## Conflicts of interest statement

All authors declare that there are no conflicts of interest.
